# Control of (pre)-analytical aspects in immunoassay measurements of metabolic hormones in rodents

**DOI:** 10.1530/EC-18-0035

**Published:** 2018-03-14

**Authors:** Maximilian Bielohuby, Martin Bidlingmaier, Uwe Schwahn

**Affiliations:** 1 Sanofi-Aventis Deutschland GmbHR&D, Industriepark Höchst, Frankfurt, Germany; 2 Endocrine Research LaboratoriesMedizinische Klinik und Poliklinik IV, Klinikum der Universität München, Munich, Germany

**Keywords:** variability, mice, immunoassay, ELISA, insulin, plasma hormones, assay performance, assay validation, multiplex, metabolism, endocrine factors

## Abstract

The measurement of circulating hormones by immunoassay remains a cornerstone in preclinical endocrine research. For scientists conducting and interpreting immunoassay measurements of rodent samples, the paramount aim usually is to obtain reliable and meaningful measurement data in order to draw conclusions on biological processes. However, the biological variability between samples is not the only variable affecting the readout of an immunoassay measurement and a considerable amount of unwanted or unintended variability can be quickly introduced during the pre-analytical and analytical phase. This review aims to increase the awareness for the factors ‘pre-analytical’ and ‘analytical’ variability particularly in the context of immunoassay measurement of circulating metabolic hormones in rodent samples. In addition, guidance is provided how to gain control over these variables and how to avoid common pitfalls associated with sample collection, processing, storage and measurement. Furthermore, recommendations are given on how to perform a basic validation of novel single and multiplex immunoassays for the measurement of metabolic hormones in rodents. Finally, practical examples from immunoassay measurements of plasma insulin in mice address the factors ‘sampling site and inhalation anesthesia’ as frequent sources of introducing an unwanted variability during the pre-analytical phase. The knowledge about the influence of both types of variability on the immunoassay measurement of circulating hormones as well as strategies to control these variables are crucial, on the one hand, for planning and realization of metabolic rodent studies and, on the other hand, for the generation and interpretation of meaningful immunoassay data from rodent samples.

## Introduction

After the discovery, in 1959, of the radioimmunoassay which enabled the measurement of insulin in human plasma (1), circulating metabolic hormones such as insulin (2) or growth hormone (3, 4) were quickly investigated for research purposes in laboratory rodents. The replacement of radioactivity by an enzymatic label in 1971 by Engvall and Perlmann (ELISA) (5) as well as by van Weemen and Schuurs (EIA) (6), and the associated increased handling convenience led to a boost in using immunoassays and ever since they have arrived as standard tools in science. Today, the analysis of metabolic hormones by immunoassay is an essential diagnostic tool in medicine. In parallel, immunoassay measurements of circulating parameters have been crucial for an overwhelming number of scientific discoveries in metabolic diseases. When measuring hormones in laboratory rodents, the paramount aim usually is to generate scientific data rather than to acquire diagnostic information. Especially after the identification of a novel circulating hormone or after the recognition of novel functions of a previously known parameter, the interest to measure this specific parameter in the circulation of rodents may arise or may experience a revival among a broader scientific community. Immunoassays are particularly suitable to serve this demand for a number of reasons. If appropriate antibodies reliably recognizing the antigen of interest are available, larger batches of immunoassays are relatively easy to produce in a short amount of time. In addition, the technical procedures to conduct immunoassays are established in most research labs and many groups have access to the required analytical equipment. It is important to distinguish between immunoassays installed for diagnostic purposes in humans and immunoassays used to detect circulating metabolic parameters in rodents for research purposes. The immunoassay classification ‘for research purposes only’ allows assay manufacturers to quickly provide the necessary tools to the research community without the need for exhaustive assay validation processes, which are typically required for diagnostic assays in human medicine. At the same time, the lack of thorough validation processes for novel immunoassays due to the need for timely solutions also comes with an important limitation: the quality of ‘research immunoassays’ is often worse compared to immunoassays used in diagnostic laboratory medicine. This topic has also recently been highlighted in an editorial discussing immunoassays for the measurement of (rodent) steroid hormones (7). It has perspicaciously been warned that using commercially available rodent immunoassays without implementing the traditional controls, such as spiked samples, mixing experiments and serial dilutions can seriously impact the quality of research data. The author also raised the awareness that self-generated data from specific samples used in the experiments should be used to describe the performance of an immunoassay rather than reporting performance characteristics provided by the manufacturer in the ‘instructions for use’ (IFU) (7).

The primary aim of this review is to bring this issue to the attention of researches with the goal to also provide some guidance on how to avoid common pitfalls. Furthermore, recommendations are given how to perform a basic validation of novel immunoassays for measurement of metabolic hormones in rodents. Finally, practical measurement examples should increase the awareness for the potential introduction of unwanted variability during the pre-analytical and the analytical phases.

## Sources of variability in immunoassays

By nature, every analytical method is subject to variability. In addition to the biological variability usually being the desired readout of immunoassay analyses in research, pre-analytical and analytical variability also affect the measurement readout in immunoassays. Pre-analytical and analytical variability in this context relate to the phenomenon that measurement readouts in immunoassay always need to be regarded as relative analyte concentrations (mostly relative to a calibrator curve) and thus the term ‘variability’ seeks to describe the difference between the true analyte concentration in the sample and the observed measurement result (8, 9). Both types of variability are typically less well controlled and studied in research immunoassays used for measurement of metabolic hormones in rodents compared to established immunoassays used in routine laboratory diagnostics. Pre-analytical variability describes the variability potentially introduced by differences related to sample collection, sample preparation and processing as well as sample storage (9, 10, 11, 12, 13). In contrast, analytical variability summarizes the variability introduced into a measurement result by factors that are directly connected to the analytical method, such as the choice and specificity of antibodies, assay buffers, assay design (sandwich vs competitive), matrix differences between calibrators and samples, incubation times or interference with other endogenous ligands (8, 9, 14, 15). Pre-analytical variability sometimes is regarded to be of minor importance. However, it has been estimated that the variability introduced during this phase accounts for up to 93% of the total errors encountered within the entire diagnostic process in human laboratory medicine (12). Considering that the pre-analytical phase in human medicine – despite well-known shortcomings – already has a high degree of standardization, it must be assumed that in the less well-controlled and standardized environment of many rodent studies the factor ‘pre-analytical variability’ might even more severely affect sample quality.

## Blood sampling site and effect of isoflurane anesthesia

One aspect that is relatively well known and described within the perimeter ‘pre-analytical and unwanted biological variability’ is that – depending on the analyte – a considerable amount of variability can potentially be introduced by the choice of the blood sampling method and site. Most available data and recommendations have been reported for humans and domestic animals (e.g. (16, 17, 18, 19, 20)), with significantly less data reported for rodents, particularly with respect to potential effects on circulating endocrine parameters. Not surprisingly, also in rodents the blood sampling site can affect measurement results. In this context, a solid amount of data has been reported for rodents showing that the blood sampling site and technique has a significant impact upon the measurement of many clinical chemistry parameters including hematopoietic cells, plasma glucose, liver lipids and enzymes and the presence of clots or hemolysis (21, 22, 23). For example, Hoggatt *et al*. have demonstrated in mice that sampling from central sites yields lower numbers of red and white blood cells compared with blood sampled peripherally (23). Schnell and colleagues also showed in mice that the site of blood collection plays a substantial role for measurement of many clinical chemistry parameters such as transaminases or lipases (24). Similarly, Fernández *et al*. found significant differences in 8 out of the 9 biochemical parameters studied (including transaminases, albumin, triglycerides, total cholesterol and creatinine) between submandibular vein and retrobulbar plexus blood collection in C57BL/6J mice (25). This type of systematic analysis is less often conducted in rodent studies and data are usually only reported for individual hormones. Among the reported hormones, particular attention has been given to hormones belonging to the hypothalamic-pituitary-adrenal (HPA) axis as well as to adrenal medullary hormones (26, 27, 28, 29, 30).

**Figure 1:**
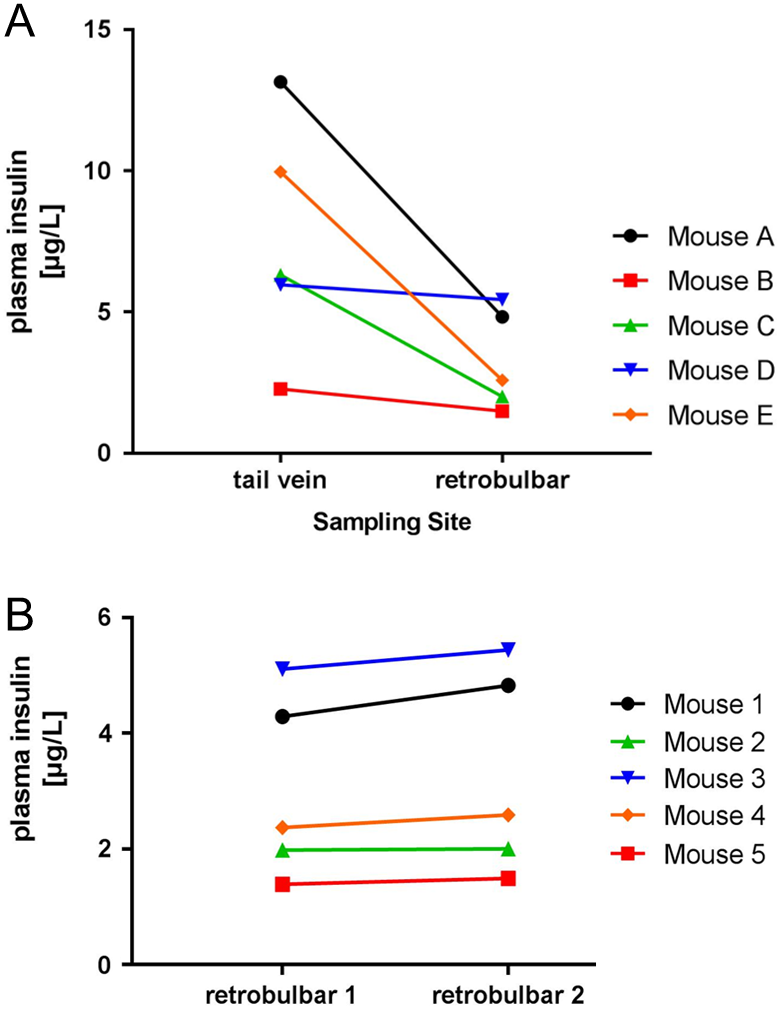
Example illustrating the effect of alternating the blood sampling site within short intervals (tail vein puncture vs retrobulbar sinus) on measured plasma insulin concentrations in five anesthetized C57BL/6J mice (A). Blood sampling sites were alternated from mouse to mouse to avoid the introduction of a systematic error. (B) Shows the expected absence of effects on measured plasma insulin concentrations in another cohort of anesthetized C57BL/6J mice when blood was sampled within short intervals from the same site (in this case from the retrobulbar sinus).

As another example of a frequently measured metabolic parameter in rodents, we here depict that the sampling site can affect the measurement results of plasma insulin in mice. Insulin concentrations were measured in samples collected either via puncture of the tail vein or from the retrobulbar sinus of adult C57BL/6J mice. During the whole sampling process, mice remained under isoflurane narcosis and the two blood samples were collected within 3 min per mouse into prefilled EDTA tubes on ice. Further details on sampling and analytical procedures can be found in the ‘Materials and methods’ section. In another cohort of 20 mice, we studied the effect of isoflurane narcosis on plasma insulin. Therefore, two blood samples were collected from the tail vein of the same mouse, one time under isoflurane narcosis in an inhalation chamber or for the subsequent sampling without anesthesia in conscious mice. Figure 1A shows that the measured plasma insulin concentrations are clearly lower in all five mice when blood was collected from the retrobulbar sinus. In contrast (and as expected), repeated sampling from the same sampling site within a short time frame did not significantly affect the measured analyte concentration (Fig. 1B). This small set of data does not claim to be a representative measure for the effect of the sampling site on plasma insulin measurements. Instead, it is intended to illustrate how quickly the alternation of the sampling site can lead to a high degree of unwanted variability in measured analyte concentrations in rodents. Thus, to avoid a potential bias in measurement results of rodent metabolic hormones within one experiment, it is very important to stay consistent with the blood sampling site, especially when comparing two or more experimental groups such as ‘compound treatment’ vs ‘vehicle treatment’ or when comparing measurement results from repeated blood samplings. Furthermore, the procedure of blood sampling should be precisely documented to help with the interpretation of measurement data also at potentially much later points in time.

**Figure 2:**
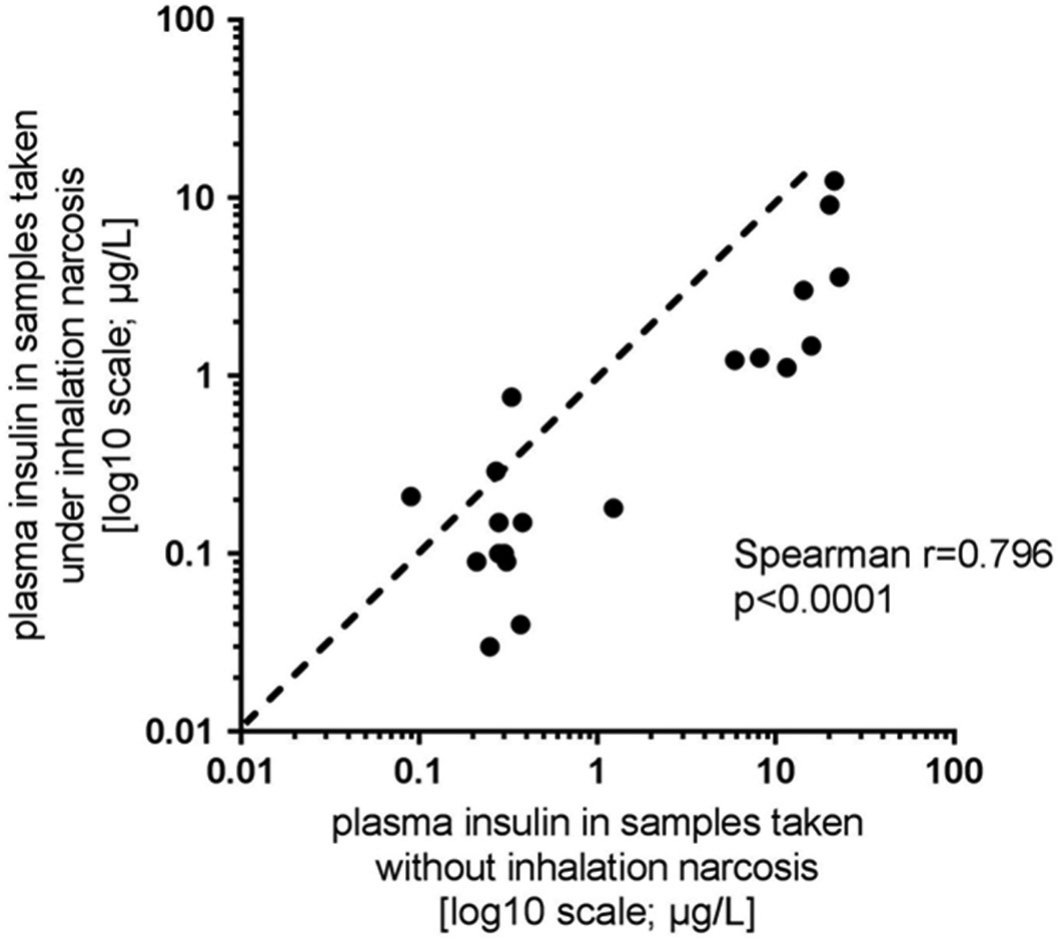
Comparison of measured plasma insulin concentrations in samples collected from the tail vein with or without prior isoflurane anesthesia in C57BL/6J mice. The anesthetic procedure before blood sampling was alternated from mouse to mouse to avoid the introduction of a systematic error. In this example, measured plasma insulin concentrations were significantly (*P* < 0.05) lower when blood was collected under isoflurane anesthesia but showed a tight and significant non-parametric correlation.

Unwanted variability during the blood sampling process may also be introduced by comparing analyte concentrations in samples that have been collected under narcosis with analyte concentrations in samples that have been collected without prior narcosis. It is well documented that anesthetics are capable to influence intestinal motility, gastric emptying and glucose metabolism (31, 32, 33). In our hands and using a small set of samples collected from the tail vein with or without isoflurane anesthesia in C57BL/6J mice, plasma insulin concentrations were significantly (*P* < 0.05) lower when blood was collected under isoflurane anesthesia. Nevertheless, plasma insulin concentrations measured in samples collected with the two different anesthesia protocols showed a tight and significant correlation (Fig. 2). The displayed data show an inhibitory effect of isoflurane narcosis on plasma insulin secretion, which is in accordance with the majority of published studies (32, 33, 34, 35, 36, 37, 38). Interestingly, Zardooz *et al*. described in male rats that the effect of isoflurane exposure on plasma insulin concentrations seems to depend on the fasting state, as they could not detect effects of the isoflurane anesthesia on plasma insulin in the fed state (39). The effect of isoflurane narcosis on plasma insulin has been found to be even more pronounced when glucose-stimulated insulin concentrations are measured, e.g. as usually done during an oral glucose tolerance test. This effect is probably best explained by the inhibitory effect of isoflurane on gastric emptying and intestinal motility, thus affecting the passage time of the orally administered glucose (33, 35, 36).

In contrast to the parameter ‘rodent insulin’, where already several groups reported clear effects of the narcosis protocol on the circulating concentrations, many other metabolic hormones in rodents unfortunately lack this type of systematic studies. It may be assumed that the type of narcosis and the sampling site can affect the measurement result of many other metabolic hormones. However, there are also examples from relatively stable circulating parameters in rodents, such as IGF-I, which seems to be only marginally affected by these variables ((40) and unpublished observations). Unfortunately, there is no strict rule that can predict if or in which direction a specific narcosis protocol or sampling site affects the analyte of interest. The easiest and best way to circumvent this potential source of unwanted biological variability in rodent studies is to stick exactly to the same sampling procedures, including the usage of the same equipment (e.g. blood sampling devices) needed to collect the sample, as well as to only compare samples from animals that underwent identical pre-treatment procedures. Of course, easily controllable biological variables such as the fasting condition before sampling, e.g. during dynamic tolerance tests, should be kept constant between experimental groups throughout the enite study. Further guidance and recommendations on this topic can also be found in recent comprehensive literature reports (41, 42).

## Sample processing

After sample collection, the ‘pre-analytical pitfall journey’ continues. Blood samples should be collected in suitable containers to obtain plain serum or alternatively in containers pre-filled with anti-coagulants such as EDTA, sodium citrate or heparin to obtain plasma. Many sources recommend the usage of either serum or heparin- or EDTA-plasma for routine biochemistry analyses (16, 43), which – in principle – are also both appropriate for most of the analyses of metabolic hormones in rodents. However, there are several analytes where serum or plasma should be preferred. For example, it has been reported that bile acids concentrations are about 60% lower when measured in heparin-plasma compared to serum (44). Trace elements like Zn or Mg can only be analyzed in serum, because in plasma, the ions form complexes with the chelating agents such as EDTA or heparin due to their bivalent nature (45). In contrast, some peptide hormones like the pituitary-derived ACTH should be measured in plasma for stability reasons (46).

It may also be useful to add an unspecific protease inhibitor to the blood sample or to pre-load the sample collection tube with such an inhibitor. This can be recommended particularly if the complete list of analytes is not yet specified at the time of sampling or if it is already known that analytes are susceptible to degradation by proteases (e.g. active GLP-1). Furthermore, it is important to check if further ‘additives’ are required to stabilize the analyte. One example for an analyte requiring a specific sampling protocol is acylated ghrelin, because here, acidification of the sample through addition of HCl is required to preserve the octanoyl group of ghrelin (47, 48). Depending on the number and stability of the analytes of interest, it might be required to collect several aliquots with different additives to obtain the optimal sample type for each analyte. Unfortunately, such an approach is often not possible in rodent studies because of the limitations in sampling volume. However, since the type of matrix as well as the sample pre-treatment conditions can have a significant impact on the immunoassay measurement result, a minimum requirement is that only data from samples which were collected and processed with the same pre-treatment protocols should be compared.

In previous experiments in rats (9), we have demonstrated that the centrifugation protocol had only limited or no effect on the measurement results of five different metabolic hormones, including insulin and GLP-1. However, the lack of effects of the centrifugation protocol on these randomly selected hormones should not to be taken as a generalized rule for all metabolic hormones in each rodent species. Again, most important is consistency in all pre-analytical procedures throughout one set of experiments. After centrifugation, serum or plasma samples must be stored in appropriate tubes (not to forget a clear and wash-off resistant sample identifier) with appropriate lids (proper sealing of the tubes can prevent evaporation/contamination of the sample). If immunoassay measurements cannot be done right away, (long-term) storage should be carried out in the freezer, ideally at −70°C or lower, again to prevent proteolytic activity and sample evaporation. Frequent freeze-thaw cycles can also seriously affect analyte concentrations (9) and thus should be avoided. If multiple analytes are to be measured using singleplex assays, it is advisable to prepare multiple aliquots of the sample.

Realizing the many steps that are necessary to obtain the final matrix in which the analyte of interest will be measured, it appears obvious that these steps can vary between different research groups and labs. Luckily, the many different possibilities of sample collection, processing and storage are not a too serious issue in preclinical rodent research as long as conditions are kept standardized and consistent within an experimental series and as long as all samples undergo exactly the same procedures before measurement. It is appeasing to realize that any potential measurement variability introduced by one or multiple specific pre-analytical procedure(s) can be considered to be of minor relevance in this context because this ‘bias’ should – in theory – affect the measurement results of all samples in the same direction and in a similar order of magnitude. Overall, the relative differences (or lack of differences) between control and observer groups within one experiment should remain constant if the above described general rules are applied. Nevertheless, it is very important to keep in mind that the pre-analytical phase has a significant impact on immunoassay measurements of rodent samples, also in order to avoid an unintended mixture of pre-analytical procedures between samples from the same experimental run. Of note, it is also likely that the selection of specific procedures during the pre-analytical phase contributes to differences in reported concentrations for the same hormones analyzed by different groups or by different labs.

## Analytical variability

Knowledge about the existence of analytical variability and a basic understanding of how to control this variable is of great importance to conduct meaningful interpretations of measured values from rodent samples. However, a comprehensive discussion of the complex topics ‘analytical variability’ and ‘measurement uncertainty’ in immunoassay measurements is beyond the scope of this review, and readers may be referred to more detailed literature for further insights (49, 50, 51, 52). Instead, the following section will summarize some considerations, which are of importance in the context of immunoassay measurement of metabolic hormones in rodents.

## Intra- and inter-assay variability in rodent immunoassays

Although the quality criteria for immunoassays used ‘for research purposes only’ (RUO) obviously can be less strict compared to immunoassays used for clinical diagnostics in human, also immunoassays used for the measurement of metabolic hormones in rodents should – in principle – follow the same guidelines and recommendations. Among others, the Clinical and Laboratory Standards Institute published recommendations with respect to acceptable limits of detection and quantification, imprecision, linearity and recovery (53, 54). The chosen analytical method (e.g. mass spectrometry or immunoassay) can significantly affect the measurement result, but also different types of the same method, e.g. sandwich-type or competitive immunoassays or assays from different manufacturers for the same analyte, likely will result in differences of reported absolute concentrations. Lack of standardization or harmonization has been recognized as one of the most relevant issues in diagnostic procedures used in clinical medicine. However, the lack of standardization is even more common in the less regulated market of RUO reagents. For example, we recently have shown considerable differences in assay readouts when IGF-I was measured in serum of mice with two different mouse-specific IGF-I immunoassays (9). Similar examples of a significant variability for the same analyte between rodent assays from different vendors are documented throughout literature. For the purpose of this review, and using the analysis of FGF-15 in mice as one example, here we illustrate the significant performance differences between immunoassays of different vendors (Table 1). To overcome such discrepancies for assays used in clinical diagnostics in humans, standardizing and harmonizing the preparations of the reference materials, using a single, recombinant calibrator for all assays of a specific analyte and reporting in the same mass units for each analyte have been recommended (55, 56). The shortcomings, but sometimes also the success of such efforts to standardize or harmonize assays in clinical labs is reflected by the results from external quality assessment schemes (EQAS), in which to participate is mandatory for clinical laboratories in most countries. This type of external quality control, however, is rarely performed for rodent research immunoassays. Luckily, the quantitative differences between assays from different manufacturers are of limited importance in most basic rodent studies as long as all samples from an experimental series have been measured in the same batch of immunoassays from the same provider – ideally in the same analytical run. In contrast to human diagnostics, where the measured concentration of an analyte is often interpreted in relation to absolute cut-off values or to analyte-specific age- and sex-matched reference intervals (and thus directly contributes to the clinical interpretation of a patient’s disease state), researchers investigating metabolic hormones in rodents are usually more interested to obtain the ‘relative changes’ of the analyte. ‘Relative’ in this context means that differences in measured concentrations are compared between an observer group and the corresponding control group. However, as soon as the analytical method to measure rodent hormones in a laboratory is being changed or data from one lab are being compared to those obtained in another lab using a different kit, the methodological differences leading to differences in absolute concentrations reported need to be taken into account.

**Table 1:** Displays some basic performance characteristics of an exemplary assay comparison between immunoassays from two commercial vendors, both measuring FGF-15 in mouse plasma.

	Vendor A	Vendor B
Precision (intra-assay CV) (%)	14.7	4.1
Recovery at a concentration range in the lower third of the calibration curve (%)	380	60
Recovery at a concentration range in the middle third of the calibration curve (%)	260	58
Recovery at a concentration range in the upper third of the calibration curve (%)	n.d.	54

Intra-assay variability was determined by 6-fold analysis, recovery was analyzed as ‘expected over observed’ concentrations using recombinant mouse FGF-15 from an external provider. Analytes were added at the low, medium and upper end of the calibration curve.

n.d.: not determined.

In addition to the above-mentioned ‘between-assay variability’ – which is attributable to differences in the assay components provided by the manufacturer and completely independent from the operator using the assay – systematic and random errors in the actual lab work can also contribute to ‘between-assay variability’, but also to ‘within-assay’ variability. Such variability is an inherent feature of any analytical procedure and relates to random variability or systematic mistakes in an operators performance of the assay, but also to variability in environmental conditions like temperature, humidity as well as in the technical performance of instruments like pipettes, plate shakers or washers. This inherent variability is often summarized as ‘coefficient of variability’ (% CV) or ‘measurement uncertainty’. Within one immunoassay run, such variability can be controlled by measuring all samples in duplicate or triplicate, a measure which – although expensive – should also be applied to rodent samples. Although the maximum acceptable degree of variability depends on the specific analyte and the specific research question, it is a common recommendation that the CV% for a duplicate analysis should not exceed 15% otherwise the measurement should be repeated. In case the available sample volume is too low, and provided that the immunoassay for the desired analyte is sensitive enough, one might consider diluting the sample with a suitable diluent in order to achieve duplicate measurements or to repeat the measurement. The impact of environmental conditions is much harder to predict and to control. However, it is recommended to monitor and document some of the basic conditions during an assay run to allow retrospective identification of factors negatively affecting assay performance. In the clinical laboratory, the concept of internal quality control samples analyzed with each assay has been implemented to control between-assay variability. Manufacturers of rodent assays sometimes provide such control samples. However, it should be carefully examined by each user of the assay if these quality control samples are suitable to monitor assay performance in a concentration range, which is meaningful to the outcome of a rodent study. Most assays perform comparably well in the mid-range of the calibration curve, while the performance is less reliable toward the lower or upper end of the standard curve concentration ranges, which might be very relevant for a scientific conclusion from an experiment. It should also be checked whether the quality control material provided with an assay mirrors ‘real samples’ in terms of matrix. It is common that so-called ‘control samples’ just are made of one of the calibrators. It depends on the matrix of the real samples whether such an approach is sufficient to monitor assay quality. To control assay performance over a larger series of immunoassay plates it might be advisable to have your own ‘control sample’ running with each assay. Such samples can be produced from leftovers samples from previous experiments and should be separated into a sufficient number of aliquots to allow measuring one of the ‘real samples’ with each assay. This allows calculation of the CV% over the measurement series, thereby giving an estimate of the uncertainty associated with the generation of the data.

## Basic assay validation steps for rodent immunoassays

It is fascinating to realize that nature has implemented such a complex set of regulatory factors controlling metabolism and new, previously unknown circulating metabolic hormones are discovered in periodic intervals. In addition, known hormones can also become a ‘hot-topic’ with the need to develop novel or more sensitive immunoassays if research reveals a previously unrecognized (maybe broader) impact of this specific hormone in other relevant scientific areas such as metabolic research. Examples for recently discovered or recognized metabolic hormones include Asprosin, the glycoprotein CHI3L1 (chitinase-3-like protein 1), GDF15, Phoenixin or members of the fibroblast growth factor family (57, 58, 59, 60, 61, 62, 63, 64, 65, 66, 67, 68, 69). In order to also offer the required research tools, providers sell analyte-specific immunoassays for rodents usually soon after the sequence/structure of the new hormone has become available or if a relevant sales market is expected. It is sometimes surprising to see how quickly after the discovery process particularly smaller biotech companies manage to offer immunoassays for the new analytes.

As mentioned earlier, FGF-15 is an example of a metabolic hormone that has emerged as a new, potentially interesting factor controlling body weight and glucose metabolism. Here, assay performance data of two mouse-specific FGF-15 immunoassays from two different vendors are displayed for the parameters ‘precision’, ‘recovery’ and ‘linearity’. Of note, the assay manufacturers/vendors of these two mouse FGF-15 immunoassays are intentionally not named as this example is meant to increase the awareness for analytical differences between immunoassays from different sources rather than to provide recommendations for a specific mouse FGF-15 immunoassay. Table 1 illustrates for this exemplary assay comparison that the robustness of measurement results as well as the overall assay performance clearly depends on the chosen immunoassay and obviously, both assays could be further optimized. The idea of conducting this type of basic assay validation is to provide guidance in case a researcher needs to opt between two (or more) available immunoassay options for a specific analyte. Furthermore, such a basic assay validation will also yield important data to decide if the chosen, newly established immunoassay is capable to deliver robust and meaningful data. Important steps of this basic validation include the analysis of precision and linearity; the latter can for example be done with serial dilutions of the calibrators or positive controls, which should always be delivered with an immunoassay kit. Other recent examples of hormone-specific and extended, state-of-the-art assay validation efforts for novel immunoassays measuring hormones in samples from laboratory animal species can be found in the cited references (70, 71, 72, 73, 74). In addition, literature research or personal exchange during scientific meetings can be helpful to elucidate if other research groups have successfully used the (potentially novel) immunoassay to measure the analyte. In conclusion, before using a rodent immunoassay for the first time, it is highly recommended to invest time to check if others have effectively used a specific immunoassay for measurement of rodent samples and to include basic validation measurements, especially if the samples of interest are precious with only little available sample volumes. Ultimately, researchers understandably want to base their conclusions on meaningful measurement results aiming to avoid the generation of random numbers from precious rodent samples. In case the validation of a novel immunoassay is not successful or if data are not convincing, one may consider using alternative approaches for the measurement of the newly discovery hormone (e.g. LC–MS/MS).

## Multiplexed immunoassays

A remarkable trend in preclinical research involving measurement of rodent metabolic hormones is the use of so-called multiplex(-ed) assays. These assays are based on a fascinating technology offering the potential to measure multiple, up to 100 analytes (75) in a single sample, which typically needs less than 100 µL of sample volume. This technique has many advantages including the increased efficiency at a reduced expense, greater output per sample/volume ratio and a higher sample throughput (76, 77).

On the contrary, this technical approach also warrants some caution and comes with challenges. Cross-reactivity and the probability of cross-talking interferences of assay components, which is increasing exponentially with the number of analytes in a multiplex immunoassay (78), have been difficult to mitigate, limiting the assay performance, and thus potentially returning inaccurate or even false results (79). Also in human diagnostics, technical and operational challenges are limiting the routine implementation of multiplexed assays in clinical settings (80). The challenges of this technology may be exemplified with the fact that all analytes are being measured in the same matrix. However, the manufacturers of multiplex technology assays, especially for rodent panels, rarely provide data on how different matrix types affect the measurement of single analytes in their multiplex panels and assume that ‘one matrix fits all’. As mentioned earlier, ACTH is an example of a hormone for which immunoassay measurements in plasma are recommended. However, rodent-specific multiplexed panels for hormones, including ACTH, are offered, which are described to also reliably function in serum (81). Similar to singleplex immunoassays, users of rodent multiplex immunoassays should carefully evaluate if the sample matrix has an effect on the analyte measurement. Overall, the same principles used for validation of single-plexed immunoassays also apply to this technology and a basic validation should be carried out rigorously for each analyte in a new multiplex panel (80), e.g. as conducted by Sun *et al.* for the simultaneous measurement of aldosterone and testosterone in mice (73). In case that the multiplex panel is pre-designed, one may use the own validation data to decide if all analytes or only a subset of analytes can be regarded to deliver reliable data.

**Figure 3:**
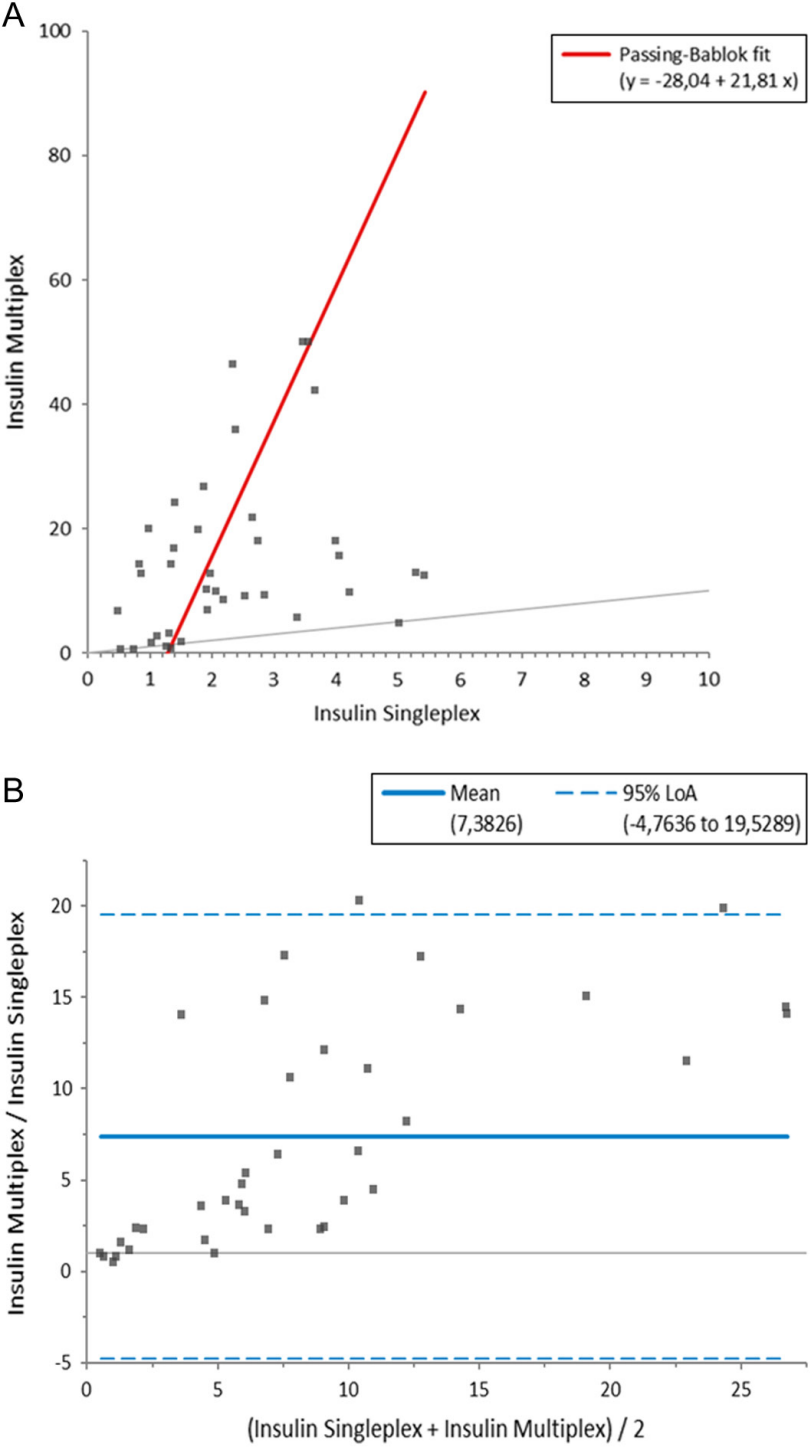
Plasma insulin was measured in the same 38 mouse samples by two different immunoassays (multiplex and singleplex). Measured concentrations showed a significant variability depending on the immunoassay. (A) Depicts the results using a Passing-Bablok regression and (B) shows the corresponding Bland-Altman plot for this assay comparison.

With respect to analytical variability Figure 3 depicts (using murine plasma insulin measurements as an example) how different the measured concentrations between multiplex and singleplex immunoassays can be. Both, Passing-Bablok regression and Bland-Altman plot show, not surprisingly, that the overall correlation is relatively weak with a comparably high fit difference. Importantly, from this analysis, one cannot determine which measurement is ‘right’ or provides the ‘true’ value in plasma, because immunoassay measurements always need to be interpreted relative to a calibration curve and do not represent an absolute quantification. However, from this example, three important conclusions can be drawn with respect to using multiplex assays: (i) measurement results can differ between multiplex and singleplex assays and neither data from multiplex nor singleplex measurements should be regarded as the ‘true’ concentrations; (ii) data should always be compared and interpreted relative to biological controls (e.g. ‘no treatment’ or ‘wild-type’ controls) measured by the same assay method and ideally also in the same experimental run; (iii) during a series of experiments, it is mandatory to stick to the same measurement method and assay throughout.

## Concluding recommendations

In summary, it is obvious that pre-analytical and analytical variability can significantly affect the reliability and reproducibility of data on metabolic hormones in rodents. In view of the innumerable spectrum of commercially available assays for the measurement of hormones in rodents, it is very important to bear in mind that the market for the ‘research-use-only’ reagents is far less strictly regulated and standardized than the market for assays used in clinical diagnostics in humans. Most of the validation of these analytical methods remains to be done by the customer, i.e., the research lab using the assays. Having this awareness at the back of one’s mind is the first crucial step for appropriate planning, and also for interpretation of metabolic studies in rodent. Notably, measures exist to control variability and to avoid frequent pitfalls, and such measures need to be implemented in an attempt to obtain high-quality data. With respect to pre-analytical variability, it is of utmost importance that all samples undergo the same standardized workflows including a careful documentation of the relevant pre-analytical steps. With respect to analytical variability, concerns have been expressed that current research immunoassays could have been pushed beyond their limits. Therefore, it is a challenge for both, assay providers and customers, to rapidly find a ‘balanced, equitable and scientifically sound solution’ (7) to improve the performance of immunoassay measurements in endocrine research involving rodent species. In the lab, one should clearly consider conducting a basic validation of an immunoassay with leftover samples before using it for the first time with precious samples. As for the pre-analytical period, also during the analytical process, all steps should follow a standardized procedure to minimize analytical variability. In case the basic immunoassay validation experiments do not provide the expected confidence in the method, contacting manufacturers or experienced colleagues may be helpful to discriminate between problems associated with the analytical method itself on the one hand and random errors in the performance of the assay on the other. Adherence to these recommendations will help to obtain reliable and meaningful data from measurements in rodent samples, ultimately reducing unpleasant surprises, resources and frustration.

## Materials and methods

### Analytical procedures related to the measurement of metabolic parameters in mice

Blood samples were collected from adult male and female C57BL/6J mice (Charles River). Mice were group-housed at room temperature in an environmentally controlled SPF-animal facility with a 12-h light-darkness cycle and had access to a standard maintenance rodent diet (Ssniff, Soest, Germany) and filtered tap water* ad libitum*. All animal experimental procedures were approved by the internal animal welfare committee as well as by German government authorities. Table 1 displays basic performance characteristics of an exemplary assay comparison between immunoassays from two commercial vendors, both commercialized for the measurement of FGF-15 in mouse serum. Intra-assay variability was determined by 6-fold analysis of mouse serum samples, and linearity was analyzed by measuring FGF-15 in serial dilutions of pooled mouse serum samples (using the diluent provided by the assay vendors). Recovery was analyzed as ‘expected over observed’ concentrations using recombinant mouse FGF-15 (% recovery = observed concentration/expected concentration × 100%). Analytes were added at the low, medium and upper end of the calibration curve. For the experiments related to different blood sampling sites, plasma insulin concentrations were measured in samples collected either via puncture of the tail vein or from the retrobulbar sinus of mice. During the whole sampling process, mice remained under isoflurane narcosis and the two blood samples were collected within 3 min per mouse. The ‘first/starting’ sampling site was alternated after each mouse to avoid the introduction of a timely bias. In another cohort of 20 mice, the effect of isoflurane narcosis on plasma insulin was studied. Therefore, two blood samples were collected from the tail vein of the same mouse, one time under isoflurane narcosis in an inhalation chamber or for the subsequent sampling without anesthesia in conscious mice (again alternating the timing of the procedure from mouse to mouse). All blood samples were collected with plastic capillaries coated with sodium heparin (SC-Sanguis Counting GmbH, Nümbrecht, Germany) into prefilled EDTA tubes on ice. After the collection process, samples were routinely and uniformly processed and stored in Nunc cryotubes (Thermo Fisher Scientific GmbH) at −80°C until analysis. All plasma samples underwent only a single freeze-thaw cycle and were analyzed in the same run of an immunoassay specific for the measurement of rodent insulin (mouse/rat insulin, Meso Scale Discovery, Gaithersburg, MD, USA). Results are depicted in Figs 1 and 2.

For the comparison of insulin single vs multiplexed immunoassays (Fig. 3), 38 plasma samples from mice were measured in duplicate and in parallel in two different mouse insulin assays (multiplex assay: Merck Millipore, Milliplex MAP Kit, Mouse Metabolic Magnetic Bead Panel, MMHMAG-44K; singleplex assay: Meso Scale Discovery, Mouse Insulin Assay, K152BZC-2). The comparison between the two immunoassay measurement methods (Passing-Bablok regression and Bland-Altman plot) has been carried out using the software Analyse-It (Analyse-it Software, Ltd., Leeds, United Kingdom, Version 4.80.2).

## Declaration of interest

Max Bielohuby and Uwe Schwahn are employees of Sanofi-Aventis Deutschland GmbH.

## Funding

This work did not receive any specific grant from any funding agency in the public, commercial, or not-for-profit sector.
